# The Changing Impact of Human Cytomegalovirus Serology and Infection on Patient Outcome After Allogeneic Hematopoietic Stem Cell Transplantation: An Italian Prospective Multicenter Survey in the Era of Letermovir Prophylaxis

**DOI:** 10.1093/ofid/ofaf233

**Published:** 2025-04-18

**Authors:** Corrado Girmenia, Patrizia Chiusolo, Giovanni Marsili, Alfonso Piciocchi, Maria Caterina Micò, Raffaella Greco, Gaetana Porto, Federica Galaverna, Francesca Bonifazi, Ilaria Cutini, Michele Malagola, Stefania Bramanti, Alessandro Busca, Angelo Michele Carella, Alessandra Carotti, Anna Paola Iori, Francesco Onida, Roberto Bono, Elisabetta Terruzzi, Adriana Vacca, Amelia Rinaldi, Irene Maria Cavattoni, Alessandra Picardi, Maura Faraci, Tiziana Lazzarotto, Fausto Baldanti, Pierangelo Clerici, Luca Castagna, Massimo Martino, Fabio Ciceri

**Affiliations:** Dipartimento di Ematologia, Oncologia, e Dermatologia, Azienda Ospedaliera Universitaria Policlinico Umberto I, Sapienza University of Rome, Rome, Italy; Sezione di Ematologia, Dipartimento di Scienze Radiologiche ed Ematologiche, Università Cattolica del Sacro Cuore, Rome, Italy; Fondazione Gruppo Italiano Malattie EMatologiche dell’Adulto, Rome, Italy; Fondazione Gruppo Italiano Malattie EMatologiche dell’Adulto, Rome, Italy; Dipartimento di Oncologia ed Ematologia, Ospedale Papa Giovanni XXIII, Bergamo, Italy; Hematology and Bone Marrow Transplant Unit, Istituto di Ricovero e Cura a Carattere Scientifico San Raffaele Hospital, Milan, Italy; University Vita-Salute San Raffaele, Milano, Italy; Hematology and Stem Cell Transplantation and Cellular Therapies Unit, Department of Hemato-Oncology and Radiotherapy, Grande Ospedale Metropolitano “Bianchi-Melacrino-Morelli,” Reggio Calabria, Italy; Department of Pediatric Hematology/Oncology, Cell and Gene Therapy, Istituto di Ricovero e Cura a Carattere Scientifico Bambino Gesù Children's Hospital, Rome, Italy; Istituto di Ematologia “Seràgnoli,” Istituto di Ricovero e Cura a Carattere Scientifico Azienda Ospedaliero–Universitaria di Bologna, Bologna, Italy; Struttura Organizzativa Dipartimentale Terapie Cellulari e Medicina Trasfusionale, Azienda Ospedaliera–Universitaria Careggi, Florence, Italy; Hematology, University of Brescia and Program of Cellular Therapies and Research in Hematology, Azienda Socio Sanitaria Territoriale Spedali Civili, Brescia, Italy; Department of Oncology/Hematology, Istituto di Ricovero e Cura a Carattere Scientifico Humanitas Research Hospital, Milan, Italy; Struttura Semplice Trapianto Allogenico Cellule Staminali, Azienda Ospedaliera Universitaria Città della Salute e della Scienza di Torino, Turin, Italy; Unità Operativa Complessa Ematologia e Centro Trapianti, Fondazione Istituto di Ricovero e Cura a Carattere Scientifico Casa Sollievo della Sofferenza, San Giovanni Rotondo, Italy; Programma Trapianto, Struttura Complessa Ematologia, Azienda Ospedale Università di Perugia, Perugia, Italy; Dipartimento di Ematologia, Oncologia, e Dermatologia, Azienda Ospedaliera Universitaria Policlinico Umberto I, Sapienza University of Rome, Rome, Italy; Struttura Complessa Ematologia, Ospedale Fatebenefratelli Sacco, Milan, Italy; Centro Trapianti di Midollo, Fondazione Istituto di Ricovero e Cura a carattere Scientifico Cà Granda, Ospedale Maggiore Policlinico, Milan, Italy; Bone Marrow Transplant Unit, Azienda Ospedaliera Ospedali Riuniti Villa Sofia Cervello, Palermo, Italy; Hematology Division, Fondazione Istituto di Ricovero e Cura a carattere Scientifico San Gerardo dei Tintori, Monza, Italy; Struttura Complessa Ematologia e Centro Trapianto di Midollo Osseo Presidio Ospedaliero, Presidio Ospedaliero Armando Businco, Azienda di Rilievo Nazionale ed Alta Specializzazione G Brotzu, Cagliari, Italy; Ematologia e Centro Trapianto di Midollo Osseo, Azienda Ospedaliero–Universitaria di Parma, Parma, Italy; Ematologia e Centro Trapianti di Midollo, Comprensorio Sanitario di Bolzano, Azienda Sanitaria Alto Adige, Bolzano, Italy; Transplant Program Azienda Ospedaliera di Rilievo Nazionale Cardarelli, Naples, Italy; Dipartimento di Biomedicina e Prevenzione, Università di Roma Tor Vergata, Rome, Italy; Hematopoietic Stem Cell Transplantation Unit, Department of Hemato-Oncology, Istituto di Ricovero e Cura a Carattere Scientifico Istituto G. Gaslini, Genoa, Italy; Microbiologia, Istituto di Ricovero e Cura a Carattere Scientifico Azienda Ospedaliero–Universitaria di Bologna, Bologna, Italy; Department of Medical and Surgical Sciences, University of Bologna, Bologna, Italy; Department of Clinical, Surgical, Diagnostic and Pediatric Sciences, University of Pavia, Pavia, Italy; Microbiology and Virology Unit, Fondazione Istituto di Ricovero e Cura a Carattere Scientifico Policlinico San Matteo, Pavia, Italy; Microbiology Unit, Azienda Socio Sanitaria Territoriale Ovest Milanese, Hospital of Legnano, Milan, Italy; Bone Marrow Transplant Unit, Azienda Ospedaliera Ospedali Riuniti Villa Sofia Cervello, Palermo, Italy; Hematology and Stem Cell Transplantation and Cellular Therapies Unit, Department of Hemato-Oncology and Radiotherapy, Grande Ospedale Metropolitano “Bianchi-Melacrino-Morelli,” Reggio Calabria, Italy; Hematology and Bone Marrow Transplant Unit, Istituto di Ricovero e Cura a Carattere Scientifico San Raffaele Hospital, Milan, Italy; University Vita-Salute San Raffaele, Milano, Italy

**Keywords:** allogeneic hematopoietic stem cell transplant, epidemiology, human cytomegalovirus, letermovir prophylaxis, survival

## Abstract

**Background:**

In the letermovir primary prophylaxis (LET-PP) era, the epidemiology of human cytomegalovirus infection (HCMV-i) in allogeneic hematopoietic stem cell transplant (allo-HSCT) recipients has changed.

**Methods:**

We prospectively evaluated incidence and risk factors for clinically significant (CS) HCMV-i at 180 days from transplant and 1-year overall survival in 1310 allo-HSCTs performed from January 2021 to March 2022 according to LET-PP use.

**Results:**

The cumulative incidence of CS-HCMV-i at 100 and 180 days from transplant was 3.8% and 16%, respectively, in patients who received LET-PP, and 14% and 17% in patients who did not. Variables associated with increased risk of CS-HCMV-i in patients who received LET-PP included transplant from an HCMV-seronegative donor, transplant from a donor other than matched related, >20 days to engraftment, and acute graft-versus-host disease (GVHD). Transplant in HCMV-seropositive recipients was associated with increased risk of CS-HCMV-i in patients who did not receive LET-PP. One-year overall survival after transplant was 81.1%. Acute leukemia, disease not in remission at transplant, Eastern Cooperative Oncology Group performance status >1, >20 days to engraftment, acute GVHD, CS Epstein-Barr virus DNAemia, gram-negative bacteremia, and invasive fungal disease were associated with increased mortality in patients who received LET-PP. HCMV recipient seropositivity, Hematopoietic Cell Transplantation Comorbidity Index score ≥3, and gram-negative bacteremia were associated with increased mortality in patients who did not receive LET-PP.

**Conclusions:**

In patients who received LET-PP, recipient/donor serology no longer correlates with early CS-HCMV-i whereas it still predicts late CS-HCMV-i as well as risk of CS-HCMV-i in patients who did not receive LET-PP. Donor serology, CS-HCMV-i and HCMV disease no longer impact survival in allo-HSCT recipients who receive LET-PP.

**Clinical Trials Registration**. NCT04412811.

## Teaser

This prospective study on the epidemiology of human cytomegalovirus infection (HCMV-i) in allogeneic hematopoietic stem cell transplant recipients in the letermovir prophylaxis era redefines the risk factors of HCMV-i and outlines a new stratification in the risk of mortality from transplantation.

Human cytomegalovirus infection (HCMV-i) is historically a major cause of morbidity and mortality in allogeneic hematopoietic stem cell transplant (allo-HSCT) recipients [1–8]. With the availability of letermovir primary prophylaxis (LET-PP) in HCMV-seropositive recipients, the risk of HCMV-i and disease, particularly during the early (0–100 days) posttransplant phase, has dramatically declined, but late HCMV-i after LET-PP discontinuation represents a challenging phenomenon [1, 2, 9, 10]. Indeed, in the letermovir era with new transplant platforms, the risk factors of HCMV-i and disease need to be redefined, and variables impacting the overall survival of allo-HSCT patients need to be reconsidered.

This prospective observational study aimed to identify the current incidence and risk factors of HCMV-i and disease in the different allo-HSCT subpopulations in the letermovir era. Factors currently affecting overall survival at 1 year from transplant are also evaluated. These data provide the basis for promoting updated direct efforts in risk stratification and management of HCMV-i in the allo-HSCT population in the LET-PP era.

## METHODS

### Study Design

The study was sponsored by the Italian Stem Cell Transplantation Network (Gruppo Italiano Trapianto di Midollo Osseo [GITMO]) and the Italian Association of Clinical Microbiologists (Associazione Microbiologi Clinici Italiani [AMCLI]) and was named the CYTOALLO GITMO-AMCLI Survey. It was a prospective epidemiological study involving 42 of the 72 total GITMO Italian centers active for allogeneic transplant activity during the study period.

### Patients

The study included patients receiving an allo-HSCT from 1 January 2021 to 31 March 2022; patients were followed for 1 year after allo-HSCT. Study start time could differ among centers, but consecutive transplants were enrolled in each center. No transplant center mentioned patients who refused to be included in the study. The results of this study were reported according to the STROBE (Strengthening the Reporting of Observational Studies in Epidemiology) statement [11]. The study was approved by the ethical committee of each center and informed consent was obtained from the patients.

### Data Collection

Variables included patients characteristics, diagnosis and phase of the underlying disease, prior HSCT, HCMV-i and diseases documented within the 3 months before transplant, prolonged neutropenia (polymorphonuclear leucocytes <500/μL cells for at least 7 days) during the month before transplant, HCMV serology of recipients and donors, Eastern Cooperative Oncology Group (ECOG) performance status at transplant, Hematopoietic Cell Transplantation Comorbidity Index (HCT-CI) score, stem cell donor, stem cell source, pretransplant conditioning regimen, use of T-cell depletion in vivo with antithymocyte globulin or ex vivo with cell manipulation, posttransplant cyclophosphamide as prophylaxis of graft-versus-host disease (GVHD), antiviral prophylaxis, duration of preengraftment neutropenia, development of acute GVHD, HCMV DNAemia detection, clinically significant HCMV-i requiring antiviral therapy (CS-HCMV-i), HCMV end-organ diseases, antiviral treatments, Epstein-Barr virus (EBV) infections requiring preemptive therapy, microbiologically documented bacterial and fungal infections, survival at 12 months from transplant, and causes of death.

### Definitions

HCMV-i and disease were defined according to international definitions of the Disease Definitions Working Group of the Cytomegalovirus Drug Development Forum [12]. In particular, HCMV-i was defined as detection of nucleic acid in blood (HCMV DNAemia) with values above the sensitivity limit of the local test. CS-HCMV-i was defined as HCMV-i or HCMV disease leading to antiviral treatment according to local practice.

### Study Objectives

The first purpose of this study was to prospectively investigate the epidemiology and risk factors of CS-HCMV-i and diseases in adult and pediatric patients undergoing allo-HSCT during the first 6 months from transplant according to the different types of underlying disease, transplant variables, and antiviral prophylaxis. The secondary objective of the study was to assess the factors that may impact on the overall survival at 12 months from transplant.

### Analyses

A separate analysis of CS-HCMV-i epidemiology and risk factors and factors associated with 1-year survival was performed for patients who received LET-PP and those who did not. The cumulative incidence of CS-HCMV DNAemia at 6 months posttransplant was calculated considering infection-free death as a competing risk, with only the first episodes of HCMV DNAemia included in the analysis. The cumulative incidence of late (>100 days) CS-HCMV DNAemia at 6 months posttransplant was calculated considering infection-free death and early CS-HCMV-i as competing risks. Cumulative incidence was assessed across various factors, including the underlying disease type and status, stem cell donor and source, pretransplant conditioning, recipient and donor HCMV serology, history of HCMV infection before transplant, and occurrence of acute GVHD. Multivariate analyses were conducted using Fine-Gray regression models for CS-HCMV-i and Cox proportional hazards regression models for 1-year posttransplant survival outcomes. In both models, all variables that reached statistical significance (*P* < .05) in the univariate analysis were included.

Survival probabilities at 1-year posttransplant were estimated using the Kaplan-Meier method, with log-rank tests applied for univariate comparisons. CS-HCMV-i, EBV DNAemia, acute GVHD, invasive fungal disease, gram-negative bacteriemia, and use of HCMV-specific immunoglobulins in prophylaxis were included as time-dependent covariates in the regression models to assess their impact only during the period following the onset. All statistical analyses were performed using R version 4.3.2 software. The CYTOALLO GITMO-AMCLI Survey is registered with ClinicalTrials.gov (NCT04412811).

## RESULTS

### Patient Characteristics

Overall, 1310 allo-HSCTs from 42 centers were prospectively included in the study. Demographic and patient characteristics at transplant are shown in Table 1. Fourteen centers enrolled 164 patients aged ≤18 years. Most of the patients were affected by acute leukemia (59.2%); 51.6% of transplants were from a human leukocyte antigen–matched related or unrelated donor while the remaining 48.4% of transplants were from haploidentical or mismatched related or unrelated donors. Overall, 879 (67.1%) HCMV-seropositive patients received LET-PP.

**Table 1.** Patients and Transplant Characteristics

Characteristic	Total Patients (n = 1310)	Letermovir Prophylaxis (n = 879)	No Letermovir Prophylaxis (n = 431)
Age, y, median (range)	52 (1–77)	55 (3–77)	37 (1–74)
Pediatric patients (age ≤18 y)	164 (12.5)	13 (1.5)	151 (35)
Male sex	784 (59.8)	513 (58.4)	271 (62.9)
Underlying disease			
Acute myeloid leukemia	557 (42.5)	404 (46.0)	153 (35.5)
Acute lymphoid leukemia	208 (15.9)	107 (12.2)	101 (23.4)
Other acute leukemias	11 (0.8)	11 (1.2)	0
Myelodysplastic syndromes	136 (10.4)	98 (11.1)	38 (8.8)
Chronic myeloproliferative	121 (9.2)	103 (11.7)	18 (4.2)
Lymphoma	143 (10.9)	104 (11.8)	39 (9.0)
Chronic lymphoid leukemia	29 (2.2)	20 (2.3)	9 (2.1)
Multiple myeloma, plasma cell leukemia, amyloidosis	16 (1.2)	10 (1.1)	6 (1.4)
Aplastic anemia	39 (3.0)	17 (1.9)	22 (5.1)
Other diseases	50 (3.8)	5 (0.6)	45 (10.4)
Phase of the underlying disease at transplant			
Malignancies in complete remission	884 (67.5)	592 (67)	292 (68)
Malignancies not in complete remission/active	246 (18.8)	183 (21)	63 (15)
Nonmalignant stable/chronic diseases	180 (13.7)	104 (12)	76 (18)
Previous HSCT			
Autologous alone	94 (7.2)	71 (8.1)	23 (5.3)
Allogeneic alone	49 (3.7)	26 (3.0)	23 (5.3)
Autologous and allogeneic	3 (0.1)	3 (0.3)	0
CS-HCMV infection/disease in the 6 mo before transplant	18 (1.4)	14 (1.6)	4 (0.9)
Prolonged neutropenia (PMN <500/μL for at least 7 d) during the month before transplant	205 (15.6)	136 (15.5)	69 (16.0)
HLA matching and donor type			
Matched related	282 (21.4)	171 (19.4)	111 (25.5)
Mismatched related	52 (4.0)	27 (3.0)	25 (5.8)
Haploidentical related	337 (25.7)	237 (27.0)	100 (23.2)
Matched unrelated	402 (30.2)	271 (30.8)	131 (30.4)
Mismatched unrelated	237 (18.1)	173 (19.7)	64 (14.9)
Stem cell source			
Bone marrow	195 (42.6)	68 (7.7)	127 (29.5)
Peripheral blood	1097 (54.7)	800 (91.0)	297 (68.8)
Cord blood	18 (2.7)	11 (1.2)	7 (1.6)
Conditioning regimen			
Myeloablative	851 (65.0)	529 (60.2)	322 (74.7)
Reduced intensity	318 (24.3)	244 (27.8)	74 (17.2)
Nonmyeloablative	141 (10.8)	106 (12.1)	35 (8.1)
T-cell depletion			
No	736 (56.2)	525 (59.7)	211 (49.0)
Yes, in vivo (antithymocyte globulin or alemtuzumab)	513 (39.2)	327 (37.2)	186 (43.1)
Yes, ex vivo (graft manipulation)	61 (4.7)	27 (30.7)	34 (7.9)
Posttransplant cyclophosphamide for GVHD prophylaxis			
No	785 (60.0)	484 (55.1)	301 (69.8)
Yes	525 (40.9)	395 (44.9)	130 (30.2)
CMV serology			
R^–^/D^–^	105 (8.6)	0	105 (24.4)
R^–^/D^+^	96 (8.9)	0	96 (22.3)
R^+^/D^–^	377 (28.2)	314 (35.7)	63 (14.6)
R^+^/D^+^	732 (54.3)	565 (64.3)	167 (38.7)
ECOG performance status at transplant			
Grade 0–1	1248 (95.3)	843 (95.9)	405 (94.0)
Grade 2–4	62 (4.7)	36 (41.0)	26 (6.0)
HCT-CI score			
0, low risk	582 (44.4)	318 (36.2)	264 (61.2)
1–2, intermediate risk	409 (31.2)	314 (35.7)	95 (22.0)
≥3, high risk	319 (24.4)	247 (28.1)	72 (16.7)
HCMV-specific immunoglobulins in prophylaxis	62 (4.7)	38 (4.3)	24 (5.6)
Early CS-HCMV, first infection, No. of cases/evaluable patients (%)	93/1310 (7.1)	33/879 (3.7)	60/431 (13.9)
Late CS-HCMV, first infection, No. of cases/evaluable patients (%)	122 /1129 (10.8)	111/796 (13.9)	11/333 (3.3)
HCMV end-organ disease	10/1310 (0.8)	7 (0.8)	3 (0.7)
Overall survival at 1 y from transplant	1063/1310 (81.1)	712/979 (81.0)	351/431 (81.4)

Data are presented as No. (%) unless otherwise indicated.

Abbreviations: CMV, cytomegalovirus; CS, clinically significant; D^+^, donor seropositive; D^–^, donor seronegative; ECOG, Eastern Cooperative Oncology Group; GVHD, graft-versus-host disease; HCMV, human cytomegalovirus; HCT-CI, Hematopoietic Cell Transplantation Comorbidity Index; HLA, human leukocyte antigen; HSCT, hematopoietic stem cell transplant; PMN, polymorphonuclear leucocytes; R^+^, recipient seropositive; R^–^, recipient seronegative.

Data on the incidence of early CS-HCMV-i (documented during the first 100 days from transplant), late CS-HCMV-i (documented after day 100 and until day 180 from transplant), end-organ HCMV diseases, and overall survival at 1 year from transplant according to the intake of LET-PP are also summarized in Table 1.

### HCMV Infections and Diseases in Patients Who Received Letermovir Prophylaxis

HCMV DNAemia was documented in 284 of 879 (32.3%) allo-HSCT recipients who received letermovir prophylaxis (first documentation within 100 days from transplant in 85 of 879 [9.7%] patients and after day 100 from transplant in 199 of 827 [24.1%] evaluable patients).

**Figure 1.**
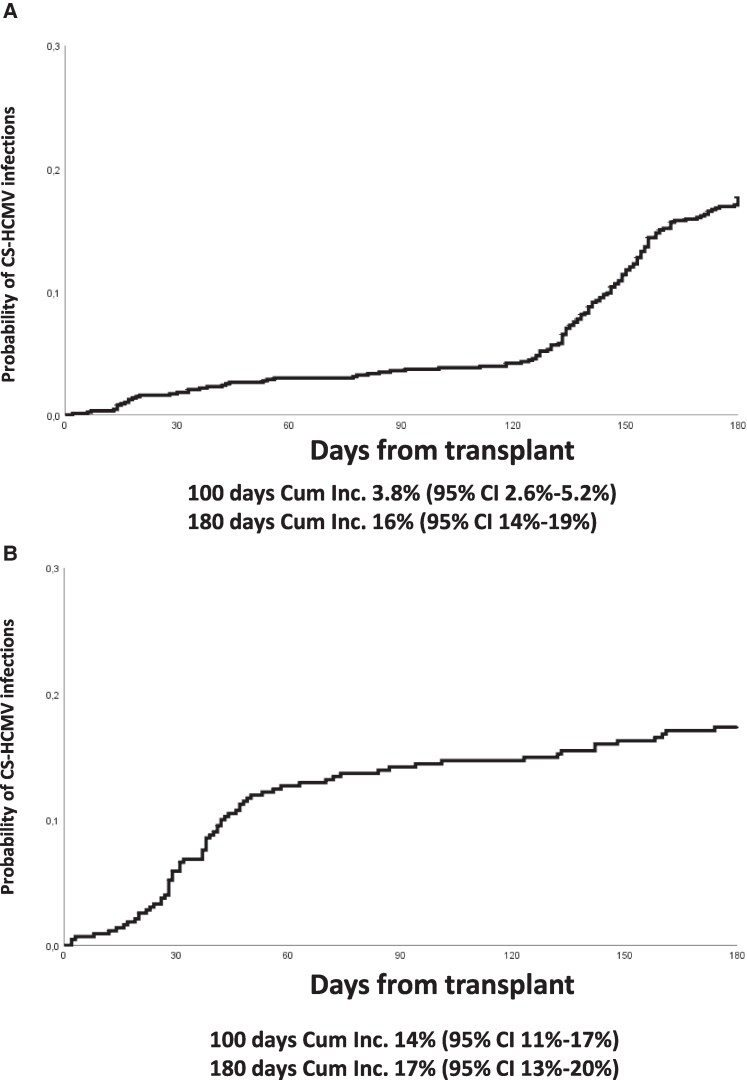
*A*, The cumulative incidence of clinically significant human cytomegalovirus (CS-HCMV) infections at 100 d and 180 d in allogeneic hematopoietic stem cell transplant (allo-HSCT) recipients who received letermovir primary prophylaxis (LET-PP) was 3.8% and 16%, respectively. The low rate of infections during the prophylaxis period was balanced by a rebound of infections in the late posttransplant phase, when prophylaxis was discontinued. *B*, The cumulative incidence of CS-HCMV infections at 100 d and 180 d in allo-HSCT recipients who did not receive LET-PP was 14% and 17%, respectively, with infections occurring mainly in the early period after transplant. Abbreviations: CI, confidence interval; CS-HCMV, clinically significant human cytomegalovirus; Cum Inc., cumulative incidence.

CS-HCMV-i occurred in 144 of 879 (16.4%) transplants. The cumulative incidence of CS-HCMV-i at 100 days and 180 days from transplant was 3.8% (95% confidence interval [CI], 2.6%–5.2%) and 16% (95% CI, 14%–19%), respectively (Figure 1*A*).

Overall, HCMV end-organ disease was documented in 7 patients who received LET-PP at 20, 126, 127, 135, 138, 152, and 162 days from transplant, respectively. They were HCMV pneumonia in 5 cases and gastrointestinal disease in 2 cases. In only 1 case, the end-organ HCMV disease was a breakthrough infection documented during LET-PP whereas in the remaining 6 cases the disease occurred after LET-PP discontinuation.

### HCMV Infections and Diseases in Patients Who Did Not Receive Letermovir Prophylaxis

HCMV DNAemia was documented in 100 of 431 (23.2%) allo-HSCT recipients who did not receive LET-PP (first documentation within 100 days from transplant in 79 of 431 [18.3%] patients and after day 100 from transplant in 21 of 393 [5.3%] evaluable patients). The cumulative incidence of CS-HCMV-i at 100 days and 180 days from transplant was 14% (95% CI, 11%–17%) and 17% (95% CI, 13%–20%), respectively (Figure 1*B*).

Overall, HCMV end-organ disease was documented in 3 patients who did not receive LET-PP at 29, 47, and 123 days from transplant, respectively. They were HCMV pneumonia in 2 cases and gastrointestinal disease in 1 case.

### Risk Factors for CS-HCMV-i in Patients Who Received Letermovir Prophylaxis

**Figure 2.**
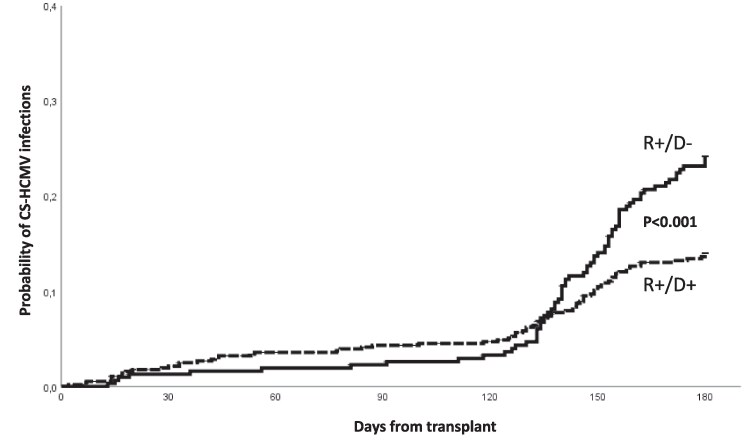
Cumulative incidence of clinically significant human cytomegalovirus (HCMV) infections at 6 mo from transplant in seropositive allogeneic hematopoietic stem cell transplant recipients who received letermovir primary prophylaxis (LET-PP) according to donor HCMV serology (*P* value univariate model). Transplant from a HCMV-seronegative donor was characterized by a significantly higher rebound of infections in the late posttransplant phase, when LET-PP was discontinued, compared to transplant from a seropositive donor. During prophylaxis, there was no difference in the incidence of clinically significant HCMV infections in the 2 groups. Abbreviations: CS-HCMV, clinically significant human cytomegalovirus; D^+^, donor seropositive; D^–^, donor seronegative; R^+^, recipient seropositive.

The risk of CS-HCMV-i occurring during the first 180 days from transplant according to demographic, underlying disease, and transplant variables are detailed in Table 2. By multivariate analysis, variables associated with increased risk of CS-HCMV-i were a previous allo-HSCT (hazard ratio [HR], 2.53 [95% CI, 1.31–4.88]; *P* = .006), a transplant from an HCMV-seronegative donor (HR, 1.63 [95% CI, 1.16–2.29]; *P* = .005), a transplant from mismatched related donor (HR, 3.39 [95% CI, 1.29–8.92]; *P* = .013) and from a haploidentical donor (HR, 2.48 [95% CI, 1.36–4.52]; *P* = .003), and >20 days’ duration to obtain engraftment (HR, 1.55 [95% CI, 1.11–2.16]; *P* = .010). Figure 2 shows the cumulative incidence of CS-HCMV-i at 6 months from transplant in patients receiving LET-PP according to the HCMV serology of the donor.

**Table 2.** Risk Factors of Clinically Significant Human Cytomegalovirus (CS-HCMV) Infection in 879 Patients Who Received Prophylaxis With Letermovir According to the Demographic, Underlying Disease, and Transplant Variables (the First CS-HCMV Infection Episode Was Considered)

Characteristic	Variable	Univariate Analysis		Multivariate Analysis	
		HR (95% CI)	*P* Value	HR (95% CI)	*P* Value
Sex	Female	1.00		…	
	Male	1.12 (.80–1.57)	.50	…	
Age (increased by 10 y)		1.00 (.99–1.01)	.86	…	
Underlying hematologic disease	Diseases other than acute leukemia	1.00		…	
	Acute leukemia	1.31 (.93–1.84)	.13	…	
Phase of the underlying disease at transplant	Complete remission	1.00		…	
	Chronic phase	1.15 (.71–1.86)	.57	…	
	No complete remission	0.86 (.55–1.33)	.49	…	
Previous HSCT	No	1.00		…	
	Previous autologous HSCT	0.58 (.27–1.25)	.16	.62 (.29–1.32)	.2
	Previous allogeneic HSCT	2.51 (1.33–4.78)	.005	2.53 (1.31–4.88)	.006
CS-HCMV infection in the 3 mo before transplant	No	1.00		…	
	Yes	2.12 (.78–5.72)	.14	…	
Recipient/donor HCMV serology	Positive/positive	1.00		…	
	Positive/negative	1.75 (1.26–2.42)	<.001	1.63 (1.16–2.29)	.005
ECOG performance status at transplant	0–1	1.00		…	
	>1	1.25 (.55–2.83)	.59	…	
HCT-CI score at transplant	0	1.00		…	
	1–2	1.03 (.70–1.52)	.89	…	
	≥3	1.13 (.75–1.70)	.56	…	
Stem cell source	Peripheral blood	1.00		…	
	Bone marrow	1.02 (.56–1.84)	.95	…	
	Cord blood	1.93 (.62–6.07)	.26	…	
Donor type	Matched related	1.00		…	
	Mismatched related	3.33 (1.28–8.67)	.014	3.39 (1.29–8.92)	.013
	Haploidentical	2.69 (1.48–4.89)	.001	2.48 (1.36–4.52)	.003
	Matched unrelated	2.41 (1.33–4.35)	.004	1.82 (.99–3.34)	.052
	Mismatched unrelated	1.99 (1.03–3.79)	.040	1.53 (.79–2.96)	.2
Conditioning regimen	Myeloablative	1.00		…	
	Nonmyeloablative/reduced intensity	0.98 (.70–1.37)	.90	…	
T-cell depletion	No	1.00		…	
	Yes	1.34 (.96–1.86)	.082	…	
Use of posttransplant cyclophosphamide as GVHD prophylaxis	No	1.00		…	
	Yes	1.25 (.90–1.75)	.19	…	
Days to engraftment	≤20 d	1.00		…	
	>20 d	1.63 (1.17–2.26)	.004	1.55 (1.11–2.16)	.010
Prophylaxis with CMV-specific immunoglobulins^a^	No	1.00		…	
	Yes	1.05 (.49–2.24)	.91	…	
Acute GVHD^a^	Grade 0–I	1.00		…	
	Grade II–IV	1.78 (1.21–2.62)	.004	1.64 (1.10–2.43)	.014
EBV DNAemia^a^	Negative or <1000 copies/mL	1.00		…	
	≥1000 copies/mL	1.85 (1.24–2.77)	.003	1.90 (1.25–2.88)	.002
Gram-negative bacteremia^a^	No	1.00		…	
	Yes	1.26 (.82–1.94)	.30	…	
Invasive fungal disease^a^	No	1.00		…	
	Yes	2.24 (1.24–4.05)	.007	1.98 (1.09–3.61)	.025

Abbreviations: CI, confidence interval; CMV, cytomegalovirus; CS, clinically significant; EBV, Epstein-Barr virus; ECOG, Eastern Cooperative Oncology Group; GVHD, graft-versus-host disease; HCMV, human cytomegalovirus; HCT-CI, Hematopoietic Cell Transplantation Comorbidity Index; HR, hazard ratio; HSCT, hematopoietic stem cell transplant.

^a^Included as time-dependent covariates in the regression model to assess their impact only during the period following the onset.

The risk factors of late CS-HCMV-i occurring from 100 to 180 days from transplant after LET-PP discontinuation were also specifically evaluated (Table 3). By multivariate analysis, variables associated with increased risk of late CS-HCMV-i were a transplant from an HCMV-seronegative donor (HR, 2.30 [95% CI, 1.55–3.40]; *P* < .001), a transplant from a haploidentical donor (HR, 3.51 [95% CI, 1.70–7.25]; *P* < .001), T-cell depletion (HR, 1.86 [95% CI, 1.19–2.91]; *P* = .006), >20 days’ duration to obtain engraftment (HR, 1.51 [95% CI, 1.02–2.22]; *P* = .038), grade II–IV acute GVHD (HR, 1.65 [95% CI, 1.08–2.51]; *P* = .021), a clinically significant EBV DNAemia (HR, 1.59 [95% CI, 1.02–2.47]; *P* = .041), and an invasive fungal disease (HR, 2.02 [95% CI, 1.05–3.89]; *P* = .036).

**Table 3.** Risk Factors of Late Clinically Significant Human Cytomegalovirus (CS-HCMV) Infection in 879 Patients Who Received Prophylaxis With Letermovir According to the Demographic, Underlying Disease, and Transplant Variables (the First CS-HCMV Infection Episode Was Considered)

Characteristic	Variable	Univariate Analysis		Multivariate Analysis	
		HR (95% CI)	*P* Value	HR (95% CI)	*P* Value
Sex	Female	1.00		…	
	Male	1.17 (.80–1.72)	.41	…	
Age (increased by 10 y)		1.01 (.99–1.02)	.40	…	
Underlying hematologic disease	Diseases other than acute leukemia	1.00		…	
	Acute leukemia	1.29 (.88–1.90)	.20	…	
Phase of the underlying disease at transplant	Complete remission	1.00		…	
	Chronic phase	1.21 (.71–2.08)	.49	…	
	No complete remission	0.91 (.55–1.49)	.70	…	
Previous HSCT	No	1.00		…	
	Previous autologous HSCT	0.64 (.28–1.45)	.28	…	
	Previous allogeneic HSCT	2.01 (.88–4.58)	.10	…	
CS-HCMV infection in the 3 mo before transplant	No	1.00		…	
	Yes	0.68 (.10–4.90)	.71	…	
Recipient/donor HCMV serology	Positive/positive	1.00		…	
	Positive/negative	2.36 (1.62–3.43)	<.001	2.30 (1.55–3.40)	<.001
ECOG performance status at transplant	0–1	1.00		…	
	>1	1.09 (.40–2.95)	.87	…	
HCT-CI score at transplant	0	1.00		…	
	1–2	1.11 (.72–1.73)	.64	…	
	≥3	1.17 (.73–1.87)	.52	…	
Stem cell source	Peripheral blood	1.00		…	
	Bone marrow	1.09 (.57–2.09)	.79	…	
	Cord blood	1.74 (.43–7.05)	.44	…	
Donor type	Matched related	1.00		…	
	Mismatched related	2.41 (.66–8.76)	.18	2.51 (.68–9.28)	.2
	Haploidentical	2.96 (1.47–5.97)	.002	3.51 (1.70–7.25)	<.001
	Matched unrelated	2.81 (1.41–5.60)	.003	1.76 (.87–3.56)	.12
	Mismatched unrelated	2.17 (1.02–4.64)	.045	1.55 (.71–3.38)	.3
Conditioning regimen	Myeloablative	1.00		…	
	Nonmyeloablative/reduced intensity	1.02 (.70–1.49)	.91	…	
T-cell depletion	No	1.00		…	
	Yes	1.58 (1.09–2.29)	.016	1.86 (1.19–2.91)	.006
Use of posttransplant cyclophosphamide as GVHD prophylaxis	No	1.00		…	
	Yes	1.30 (.88–1.90)	.18	…	
Days to engraftment	≤20 d	1.00		…	
	>20 d	1.50 (1.03–2.19)	.035	1.51 (1.02–2.22)	.038
Prophylaxis with CMV-specific immunoglobulins^a^	No	1.00		…	
	Yes	0.94 (.39–2.32)	.90	…	
Acute GVHD^a^	Grade 0–I	1.00		…	
	Grade II–IV	1.84 (1.21–2.78)	.004	1.65 (1.08–2.51)	.021
EBV DNAemia^a^	Negative or <1000 copies/mL	1.00		…	
	≥1000 copies /mL	1.89 (1.24–2.88)	.003	1.59 (1.02–2.47)	.041
Gram-negative bacteremia^a^	No	1.00		…	
	Yes	1.19 (.72–1.95)	.50	…	
Invasive fungal disease^a^	No	1.00		…	
	Yes	2.29 (1.20–4.38)	.012	2.02 (1.05–3.89)	.036

Death and early CS-HCMV infection were considered as competitive risks.

Abbreviations: CI, confidence interval; CMV, cytomegalovirus; CS, clinically significant; EBV, Epstein-Barr virus; ECOG, Eastern Cooperative Oncology Group; GVHD, graft-versus-host disease; HCMV, human cytomegalovirus; HCT-CI, Hematopoietic Cell Transplantation Comorbidity Index; HR, hazard ratio; HSCT, hematopoietic stem cell transplant.

^a^Included as time-dependent covariates in the regression model to assess their impact only during the period following the onset.

### Risk Factors for CS-HCMV-i in Patients Who Did Not Receive Letermovir Prophylaxis

**Figure 3.**
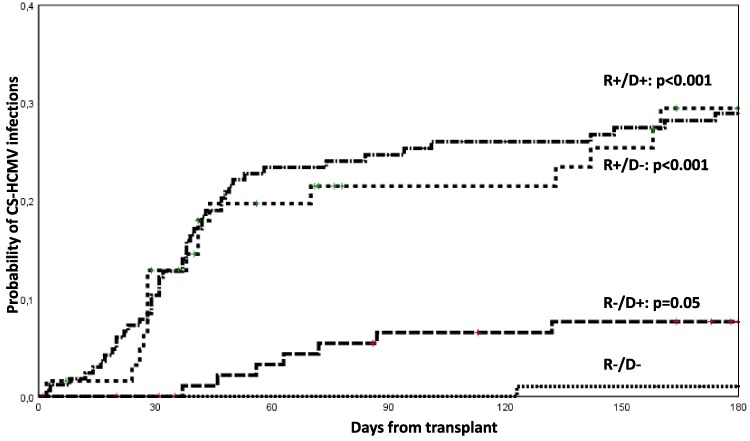
Cumulative incidence of clinically significant human cytomegalovirus (CS-HCMV) infections in allogeneic hematopoietic stem cell transplant recipients who did not receive letermovir primary prophylaxis (LET-PP) according to recipient/donor HCMV serology (*P* value univariate model). Seropositive patients who did not receive LET-PP (in most of cases being ineligible to LET-PP as they were <18 y of age) experienced several early CS-HCMV infections in the absence of LET-PP. Abbreviations: CS-HCMV, clinically significant human cytomegalovirus; D^+^, donor seropositive; D^–^, donor seronegative; R^+^, recipient seropositive; R^–^, recipient seronegative.

The risk of CS-HCMV-i in patients who did not receive LET-PP according to demographic, underlying disease, and transplant variables are detailed in Table 4. By multivariate analysis, variables associated with increased risk of CS-HCMV-i were age <18 years (age >18 vs <18 years: HR, 0.29 [95% CI, .16–.55]; *P* < .001) and the recipient-positive serostatus (R^+^/D^−^ vs R^−^/D^−^: HR, 30.2 [95% CI, 3.99–228], *P* < .001; R^+^/D^+^ vs R^−^/D^−^: HR, 23.8 [95% CI, 3.25–174], *P* = .002) (Figure 3). Considering the 151 pediatric patients aged ≤18 years who did not receive LET-PP, the incidence of CS-HCMV-i did not differ comparing patients with oncologic and nononcologic diseases as CS-HCMV-i occurred in 28 of 91 (30.8%) and in 19 of 60 (31.7%) patients, respectively.

**Table 4.** Risk Factors of Clinically Significant Human Cytomegalovirus (CS-HCMV) Infection in 431 Patients Who Did Not Receive Prophylaxis With Letermovir According to the Demographic, Underlying Disease, and Transplant Variables (the First CS-HCMV Infection Episode Was Considered)

Characteristic	Variable	Univariate Analysis		Multivariate Analysis	
		HR (95% CI)	*P* Value	HR (95% CI)	*P* Value
Sex	Female	1.00		…	
	Male	0.98 (.60–1.59)	.93	…	
Age (increased by 10 y)		0.97 (.96–.98)	<.001	…	
Age	<18 y	1.00		…	
	≥18 y	0.22 (.14–.37)	<.001	0.29 (.16–.55)	<.001
Underlying hematologic disease	Diseases other than acute leukemia	1.00		…	
	Acute leukemia	0.68 (.43–1.08)	.10	…	
Phase of the underlying disease at transplant	Complete remission	1.00		…	
	Chronic phase	1.46 (.80–2.64)	.21	1.26 (.69–2.30)	.4
	No complete remission	2.18 (1.23–3.85)	.007	1.57 (.87–2.84)	.14
Previous HSCT	No	1.00		…	
	Previous autologous HSCT	0.75 (.24–2.40)	.63	…	
	Previous allogeneic HSCT	1.67 (.72–3.86)	.23	…	
CS-HCMV infection in the 3 mo before transplant	No	1.00		…	
	Yes	3.52 (.86–14.4)	.079	…	
Recipient/donor HCMV serology	R^–^/D^–^	1.00		…	
	R^–^/D^+^	7.81 (.96–63.4)	.055	7.42 (.91–60.5)	.061
	R^+^/D^–^	34.8 (4.43–262)	<.001	30.2 (3.99–228)	<.001
	R^+^/D^+^	35.0 (4.82–254)	<.001	23.8 (3.25–174)	.002
ECOG performance status at transplant	0–1	1.00		…	
	>1	0.69 (.22–2.20)	.53	…	
HCT-CI score at transplant	0	1.00		…	
	1–2	0.66 (.36–1.21)	.18	0.85 (.45–1.62)	.6
	≥3	0.43 (.18–.99)	.048	0.01 (.39–2.58)	>.9
Stem cell source	Peripheral blood	1.00		…	
	Bone marrow	1.64 (1.02–2.65)	.043	0.71 (.41–1.21)	.2
	Cord blood	1.28 (.18–9.31)	.81	0.68 (.09–5.28)	.7
Donor type	Matched related	1.00		…	
	Mismatched related	1.86 (.73–4.76)	.19	…	
	Haploidentical	1.61 (.85–3.07)	.15	…	
	Matched unrelated	1.07 (.55–2.06)	.85	…	
	Mismatched unrelated	0.80 (.33–1.94)	.62	…	
Conditioning regimen	Myeloablative	1.00		…	
	Nonmyeloablative/reduced intensity	0.85 (.49–1.48)	.56	…	
T-cell depletion	No	1.00		…	
	Yes	1.37 (.85–2.19)	.19	…	
Use of posttransplant cyclophosphamide as GVHD prophylaxis	No	1.00		…	
	Yes	1.25 (.74–2.12)	.40	…	
Days to engraftment	≤20 d	1.00		…	
	>20 d	1.16 (.70–1.93)	.57	…	
Prophylaxis with CMV-specific immunoglobulins^a^	No	1.00		…	
	Yes	0.46 (.11–1.88)	.28	…	
Acute GVHD^a^	Grade 0–I	1.00		…	
	Grade II–IV	1.26 (.62–2.56)	.53	…	
EBV DNAemia^a^	Negative or <1000 copies/mL	1.00		…	
	≥1000 copies/mL	0.90 (.35–2.32)	.83	…	
Gram-negative bacteremia^a^	No	1.00		…	
	Yes	0.09 (.54–2.19)	.81	…	
Invasive fungal disease^a^	No	1.00		…	
	Yes	2.05 (.75–5.63)	.16	…	

Abbreviations: CI, confidence interval; CMV, cytomegalovirus; CS, clinically significant; D^+^, donor seropositive; D^–^, donor seronegative; EBV, Epstein-Barr virus; ECOG, Eastern Cooperative Oncology Group; GVHD, graft-versus-host disease; HCMV, human cytomegalovirus; HCT-CI, Hematopoietic Cell Transplantation Comorbidity Index; HR, hazard ratio; HSCT, hematopoietic stem cell transplant; R^+^, recipient seropositive; R^–^, recipient seronegative.

^a^Included as time-dependent covariates in the regression models to assess their impact only during the period following the onset.

### Survival

One-year posttransplant mortality was 18.9% (247 patients). Death was related to hematological disease relapse in 89 (36%) cases and to a transplant complication in 158 (64%) cases. In 52 patients (21%) the primary cause of death was an infection, and in only 1 case due to an HCMV pulmonary disease.

The overall survival at 12 months after transplant was 81.1% (95% CI, 79.0%–83.2%). It was 80.9% and 81.4% in patients who received LET-PP and in those who did not, respectively.

Variables involved with the overall survival at 1 year from transplant were separately evaluated according to LET-PP intake (Table 5). In patients who received LET-PP, a diagnosis of acute leukemia, a disease not in complete remission at the time of HSCT, an ECOG performance status >1, prolonged (>20 days) preengraftment neutropenia, grade II–IV acute GVHD, clinically significant EBV DNAemia, gram-negative bacteremia, and invasive fungal disease were factors independently associated with increased mortality rate. Use of HCMV-specific immunoglobulins administered in prophylaxis was associated with a significantly reduced mortality rate. Conversely, recipient/donor HCMV serology and CS-HCMV-i did not correlate with the patient outcome at 12 months from transplant.

**Table 5.** Variables Involved With Overall Survival at 1 Year From Transplant According to Letermovir Primary Prophylaxis Status

Characteristic	Variable	LET-PP				No LET-PP			
		Univariate		Multivariate		Univariate		Multivariate	
		HR (95% CI)	*P* Value	HR (95% CI)	*P* Value	HR (95% CI)	*P* Value	HR (95% CI)	*P* Value
Sex	Male vs female	1.21 (.88–1.65)	.24	…		0.84 (.54–1.32)	.46	…	
Age (increased by 10 y)		1.01 (1.01–1.03)	.034	1.01 (1.00–1.02)	.11	1.02 (1.01–1.03)	<.001	…	
Age >18 y vs ≤18 y		0.80 (.26–2.51)	.70	…		3.05 (1.68–5.53)	<.001	2.16 (.99–4.69)	.052
Underlying hematologic disease	Diseases other than acute leukemia	1.00		…		1.00		…	
	Acute leukemia	1.38 (1.00–1.90)	.049	1.52 (1.02–2.26)	.04	1.52 (.95–2.42)	.081	…	
Phase of the underlying disease at transplant	Complete remission	1.00		…		1.00		…	
	Chronic phase	0.85 (.50–1.44)	.55	1.20 (.67–2.14)	.5	1.00 (.56–1.81)	.99	…	
	No complete remission	1.49 (1.05–2.11)	.026	1.70 (1.13–2.54)	.010	1.25 (.69–2.26)	.45	…	
Previous HSCT	No	1.00		…		1.00		…	
	Previous autologous HSCT	0.90 (.50–1.63)	.73	…		2.08 (1.00–4.33)	.051	…	
	Previous allogeneic HSCT	1.55 (.76–3.16)	.23	…		1.47 (.64–3.40)	.36	…	
CS-HCMV infection in the 3 mo before transplant	No	1.00		…		…		…	
	Yes	1.19 (.38–3.72)	.77	…		1.31 (.18–9.39)	.79	…	
Recipient/donor HCMV serology	Negative/negative	…		…		1.00		…	
	Negative/positive	…		…		1.10 (.54–2.24)	.80	1.14 (.53–2.44)	.7
	Positive/negative	1.00		…		2.08 (1.04–4.17)	.039	1.89 (.90–3.96)	.091
	Positive/positive	0.88 (.64–1.21)	.43	…		1.43 (.78–2.64)	.25	2.36 (1.15–4.44)	.017
ECOG performance status at transplant	0–1	1.00		…		1.00		…	
	>1	2.42 (1.37–4.26)	.002	2.30 (1.28–4.11)	.005	1.88 (.91–3.91)	.089	…	
HCT-CI score at transplant	0	1.00		…		1.00		…	
	1–2	0.99 (.68–1.45)	.96	0.88 (.60–1.29)	.5	2.45 (1.44–4.16)	<.001	1.60 (.90–2.86)	.12
	≥3	1.51 (1.04–2.18)	.028	1.18 (.80–1.74)	.4	3.55 (2.09–6.04)	<.001	2.77 (.47–5.22)	.002
Stem cell source	Peripheral blood	1.00		…		1.00		…	
	Bone marrow	0.80 (.43–.47)	.47	…		0.45 (.25–.82)	.009	0.82 (.40–1.67)	.6
	Cord blood	0.92 (.23–3.72)	.91	…		2.78 (.87–8.85)	.084	3.65 (1.05–12.7)	.042
Donor type	Matched related	1.00		…		1.00		…	
	Mismatched related	1.74 (.81–3.77)	.16	…		0.48 (.11–2.06)	.32	0.40 (.09–1.80)	.2
	Haploidentical	1.14 (.74–1.76)	.55	…		1.19 (.62–2.26)	.60	1.05 (.49–2.26)	.9
	Matched unrelated	0.70 (.44–1.12)	.14	…		0.88 (.46–1.68)	.70	0.64 (.32–1.29)	.2
	Mismatched unrelated	1.04 (.65–1.67)	.87	…		2.39 (1.28–4.46)	.006	1.60 (.81–3.18)	.2
Conditioning regimen	Myeloablative	1.00		…		1.00		…	
	Nonmyeloablative/reduced intensity	0.84 (.62–1.16)	.29	…		1.67 (1.06–2.65)	.028	0.97 (.58–1.64)	>.9
T-cell depletion	No	1.00		…		1.00		…	
	Yes	0.78 (.57–1.07)	.13	…		0.61 (.39–.96)	.031	0.93 (.52–1.67)	.8
Use of posttransplant cyclophosphamide as GVHD prophylaxis	No	1.00		…		1.00		…	
	Yes	0.92 (.68–1.25)	.60	…		0.63 (.40–.99)	.043	0.96 (.49–1.87)	>.9
Days to engraftment	≤20 d	1.00		…		1.00		…	
	>20 d	1.63 (1.20–2.21)	.002	1.52 (1.11–2.08)	.009	1.39 (.87–2.21)	.17	…	
Acute GVHD^a^	Grade 0–1	1.00		…		1.00		…	
	Grade 2–4	2.09 (1.48–2.95)	<.001	1.80 (1.27–2.55)	.001	1.40 (.79–2.48)	.25	…	
HCMV-specific immunoglobulins in prophylaxis^a^	No			…		…		…	
	Yes	.12 (.02–.85)	.034	0.11 (.02–.82)	.031	0.74 (.80–3.77)	.16	…	
CS-HCMV DNAemia^a^	No	1.00		…		1.00		…	
	Yes	2.00 (1.32–3.04)	.001	0.50 (.98–2.31)	.064	0.70 (.33–1.46)	.34	…	
EBV DNAemia^a^	Negative or <1000 copies/mL	1.00		…		1.00		…	
	≥1000 copies /mL	1.91 (1.33–2.75)	<.001	1.57 (1.08–2.28)	.018	0.84 (.44–1.60)	.59	…	
Gram-negative bacteremia^a^	No	1.00		…		1.00		…	
	Yes	2.30 (1.64–3.27)	<.001	1.96 (1.38–2.78)	<.001	2.78 (1.71–4.52)	<.001	3.38 (2.00–5.72)	<.001
Invasive fungal disease^a^	No	1.00		…		1.00		…	
	Yes	2.82 (1.75–4.55)	<.001	2.72 (167–4.41)	<.001	0.70 (.33–1.46)	.34	…	

Abbreviations: CI, confidence interval; CS, clinically significant; EBV, Epstein-Barr virus; ECOG, Eastern Cooperative Oncology Group; GVHD, graft-versus-host disease; HCMV, human cytomegalovirus; HCT-CI, Hematopoietic Cell Transplantation Comorbidity Index; HR, hazard ratio; HSCT, hematopoietic stem cell transplant; LET-PP, letermovir primary prophylaxis.

^a^Included as time-dependent covariates.

In patients who did not receive LET-PP, recipient HCMV seropositivity, high HCT-CI score at transplant, cord blood transplant, and gram-negative bacteremia were factors associated with an increased mortality rate.

Out of 7 patients who received LET-PP and developed an HCMV end-organ disease, 2 died at 233 days from transplant due to the viral pulmonary infection and at 129 days due to leukemia relapse with concomitant HCMV pulmonary infection, respectively. All 3 patients who did not receive LET-PP and developed an HCMV end-organ disease survived at 1 year from transplant.

## DISCUSSION

In the pre-letermovir era, allo-HSCT recipients with positive HCMV serology were at high risk for early CS-HCMV-i resulting in increased nonrelapse mortality and decreased disease-free survival, with the most unfavorable condition being a transplant from an HCMV-seronegative donor to a seropositive recipient [6–8,13,14] In view of the above, the recipient serostatus has been considered a major criterion for donor selection: The choice of an HCMV-seronegative donor for an HCMV-seronegative recipient, and of an HCMV-seropositive donor for an HCMV-seropositive recipient, has been recommended, if possible, particularly in the setting of unrelated allo-HSCT prepared with myeloablative conditioning [1, 2].

With the advent of LET-PP, the epidemiology and clinical impact of CS-HCMV-i and diseases have significantly changed in the population of HCMV-seropositive recipients. A large phase 3 randomized, placebo-controlled trial and several real-world observational studies demonstrated that LET-PP administered during the first 100 days after transplantation in HCMV-seropositive patients is highly effective in reducing the likelihood of early CS-HCMV-i by day +100 and HCMV disease by day +200, and significant lower odds of all-cause and nonrelapse mortality beyond day 200 after allo-HSCT [9, 10]. Indeed, LET-PP in HCMV-seropositive patients is now considered the standard of care in the first 100 days after transplantation [1, 2, 15].

The CYTOALLO GITMO-AMCLI Survey is the largest prospective study conducted to date on the epidemiology of HCMV-i and disease. It provides a comprehensive and up-to-date description of HCMV reactivation and disease findings in a heterogeneous allo-HSCT population in the current LET-PP era. A valuable characteristic of this study, which included about 60% of allo-HSCTs performed in Italy in the study period, was the inclusion of complete denominator data prospectively collected for consecutive patients who received transplant at each center.

A limitation in the interpretation of the data in this study could be represented by the variability in the virological techniques (ie, blood vs plasma as matrix used), in the interpretation of the HCMV DNAemia results (HCMV DNAemia threshold for the start of preemptive therapy, interpretation of the so called “blips” during LET-PP), and in the indication for the use and timing of LET-PP in the different transplant centers. However, considering that such variabilities are expected in a real scenario, that most centers reported administering LET-PP usually from the first week and up to day 100 from transplantation (or until CS-HCMV-i), and that different virological techniques are substantially comparable in detecting HCMV infection, we do not believe that these possible variabilities could have significantly influenced the interpretation of the data.

The analysis of the epidemiology, risk factors for CS-HCMV-i and variables involved in the overall survival was conducted separately for patients who received LET-PP and for those who did not, as these populations are noncomparable in terms of age and recipient serological status as well. As we will comment further below, the use of letermovir seems nowadays crucial in defining the natural history of allo-HSCT and in influencing many clinical phenomena.

The cumulative incidence of CS-HCMV-i in seropositive patients who received LET-PP was 3.8% at 100 days and 16% at 180 days from transplant, confirming the findings observed since the early clinical trials [9, 10]—a low rate of infections during the prophylaxis period that was balanced by a rebound of infections in the late posttransplant phase, when prophylaxis was discontinued. The high rate of late CS-HCMV-i after discontinuing LET-PP may result from a delay of HCMV-specific CD4 and CD8 T-cell immune reconstitution, which is presumably related to suppression of HCMV replication and subsequent lack of exposure of the immune system to viral antigens during antiviral prophylaxis [16–20]. The phenomenon of HCMV replication inhibition during LET-PP with a rebound in infections after prophylaxis discontinuation has been also observed in a recent placebo-controlled phase 3 trial of extended duration of LET-PP [21]. In this study, after discontinuation of LET-PP at 200 days (28 weeks) following transplantation, there was an increased incidence of CS-HCMV-i until week 38 in the letermovir group, which was similar to the incidence in the placebo group at the same timepoint. Given that LET-PP results in virological suppression rather than cure, we should expect a certain number of late infections after prophylaxis anyway, thus suggesting the need for an extended monitoring of viremia. However, these infections appear to be more manageable, generally with a less adverse impact on overall transplant outcomes. The other variables associated with a significantly increased risk of CS-HCMV-i in our study—a previous allo-HSCT, a donor other than matched related, prolonged preengraftment neutropenia, grade II–IV acute GVHD, and clinically significant EBV DNAemia—are expected factors that correlate with immune recovery and the risk for various types of infection. In addition, HSCTs from seronegative donors, transplant from haploidentical donors, T-cell depletion as GVHD prophylaxis, and clinically significant EBV infections were identified as conditions specifically associated with a significant risk of late CS-HCMV-i occurring after LET-PP discontinuation. This information may be useful in identifying patients who may benefit from an extension of prophylaxis beyond 100 days after transplant. Another result of particular interest from our study was the low incidence of end-organ diseases, which were documented almost exclusively after LET-PP discontinuation and resulted in poor outcomes in only a minority of cases.

The cumulative incidence of CS-HCMV-i in patients who did not receive LET-PP was 14% at 100 days and 17% at 180 days from transplant, in most cases being documented in HCMV-seropositive recipients during the early posttransplant period. Patients aged <18 years were at higher risk of CS-HCMV-i compared to adults. This finding was likely related to the fact that adults who did not receive LET-PP were either HCMV-seronegative or seropositive but not given LET-PP as they were presumably considered at low risk for transplant-related complications due to their specific clinical and transplant characteristics. On the other hand, the seropositive population of patients ineligible to LET-PP due to age (<18 years old) experienced several early CS-HCMV-i, with a positive serostatus in the recipient being a significant risk factor for early viral infection in the absence of LET-PP. This finding supports the need to extend LET-PP indications for use to seropositive young transplant recipients.

Our study evaluated pretransplant factors and events occurring during the first 6 months from transplant that could potentially impact 1-year overall survival after allo-HSCT in the LET-PP era. For the analysis of factors correlated with survival, we also decided to carry out a separate analysis based on the use or not of LET-PP. The overall survival was 81.1% at 1 year after allo-HSCT and was comparable in the 2 populations. A diagnosis of acute leukemia, a hematologic disease not being in complete remission at transplant, a poorer performance status of the patient at transplant, the long duration of preengraftment neutropenia, grade II–IV acute GVHD, clinically significant EBV DNAemia, gram-negative bacterial infections, and invasive fungal infections were independent variables of survival in patients who received LET-PP. Of interest, the administration of HCMV-specific immunoglobulins in prophylaxis was associated with an improved overall outcome. These data should be interpreted with caution considering that the positive effect of HCMV-specific immunoglobulin prophylaxis on survival has never been reported in the literature. Furthermore, in our study and in other previous studies, HCMV-specific immunoglobulins administered as prophylaxis failed to contribute to the reduction of HCMV infections, and guidelines do not recommend its use in HSCT [1, 2]. However, it must be considered that in the letermovir era the use of HCMV-specific immunoglobulins in prophylaxis has not been evaluated in large prospective studies and it is therefore not possible to conclusively define their role in the prevention of HCMV infections.

Our study showed that in patients submitted to LET-PP recipient/donor HCMV serology, CS-HCMV-i and HCMV diseases no longer correlate with the patient outcome, contrary to what was shown in large experiences from the pre–LET-PP era [6–8]. In particular, donor serology in seropositive recipients did not impact survival, presumably due to the reduction of early CS-HCMV-i in all transplant conditions, including transplants from seronegative donors. In a recent retrospective single-center cohort study, LET-PP abrogated the mortality gap due to HCMV serostatus with a protective effect that persisted after discontinuation of primary prophylaxis [22]. All of these data may support a major change in transplant standards redefining the criteria for selecting donors for seropositive recipients [1, 2]. The preference of a seropositive donor for a seropositive patient is no longer mandatory given the lack of influence on patient outcome; however, the risk of late CS-HCMV-i in transplants with recipient/donor viral serology mismatch may still justify preferring a seropositive donor for a seropositive recipient, if possible. Our study also demonstrates that both CS-HCMV-i and disease no longer correlate with poorer outcomes in seropositive patients submitted to LET-PP. By delaying infections through LET-PP, they become easier to manage during a later phase of the transplant, which consequently mitigates issues related to infection treatment.

In the population that did not receive LET-PP, high HCT-CI score at transplant, gram-negative bacteremia, and importantly, HCMV seropositivity of the recipient were the variables associated with a significantly poorer outcome. These data, in confirming how the impact of recipient HCMV serology on survival outside of LET-PP is still relevant, further support the need to extend the indication of LET-PP to the pediatric population, for which the use of letermovir is awaiting approval.

In conclusion, our study identifies incidence and risk factors for CS-HCMV-i and disease as well as variables related to 1-year survival in allo-HSCT recipients in a LET-PP real-life scenario. Recipient/donor serology still predicts the risk of early and late CS-HCMV-i and impacts overall survival in patients who do not receive LET-PP, whereas it no longer correlates with early CS-HCMV-i but still predicts late CS-HCMV-i risk in seropositive patients who receive LET-PP. CS-HCMV-i and diseases no longer represent life-threatening complications in allo-HSCT recipients. Our study supports a change in the priority of criteria for HSCT donor selection, enabling relevant factors others than HCMV donor serology in seropositive recipients. These real-life data may be helpful in redefining some infection control strategies, particularly in the late phase of transplantation, for which it is still necessary to define virological monitoring and prevention practices.

## Notes

***Acknowledgments.*** The authors are indebted to Roberto Ricci and Eliana De Grandi for their valuable work in the data management.

***Author contributions.*** Each author named in the author byline contributed to the study design and to the data acquisition. C. G., G. M., A. Piciocchi, and F. C. have accessed and verified the data. G. M., A. P., and C. G. did the statistical analysis. C. G. wrote the first draft of the report with input from F. C. and L. C.

***Disclaimer.*** The funding source had no role in identifying statements, synthesizing results, preparing the manuscript, or the decision to submit the manuscript for publication.

***Financial support.*** This work was supported by Biotest Italy.

***Potential conflicts of interest.*** All authors: No reported conflicts of interest.

## APPENDIX

CYTOALLO GITMO-AMCLI Investigators:

- Corrado Girmenia, Anna Paola Iori, Walter Barberi, Ursula La Rocca, Dipartimento di Ematologia, Oncologia, e Dermatologia, and Guido Antonelli, Ombretta Turriziani, Matteo Rossi, Dipartimento di Medicina Molecolare, AOU Policlinico Umberto I, Sapienza University of Rome, Rome
- Simona Sica, Patrizia Chiusolo, Maria Assunta Limongiello, Sabrina Giammarco, Elisabetta Metafuni, Sezione di Ematologia, Dipartimento di Scienze Radiologiche ed Ematologiche and Maurizio Sanguinetti, Michela Sali, Dipartimento di Scienze Biotecnologiche di Base, Cliniche Intensivologiche e Perioperatorie, Università Cattolica del Sacro Cuore, Rome
- Giovanni Marsili, Alfonso Piciocchi, Fondazione GIMEMA (Gruppo Italiano Malattie EMatologiche dell’Adulto), Rome
- Maria Caterina Micò, Alessandra Algarotti, USC Ematologia and Marco Enrico Giovanni Arosio UOC Microbiologia e Virologia, ASST Papa Giovanni XXIII, Bergamo
- Raffaella Greco, Edoardo Campodonico, Giorgio Orofino, Consuelo Corti, Fabio Ciceri, Hematology and BMT Unit, San Raffaele Hospital, Milan
- Massimo Martino, Gaetana Porto, Caterina Alati, Barbara Loteta, Maria Caterina Micò, Giuseppe Console, Giuseppe Irrera, Hematology and Stem Cell Transplantation and Cellular Therapies Unit (CTMO), Department of Hemato-Oncology and Radiotherapy and Luigi Principe, Antonia Meliadò, Microbiology and Virology Laboratory, Grande Ospedale Metropolitano “Bianchi-Melacrino-Morelli,” Reggio Calabria
- Federica Galaverna, Maria Giuseppina Cefalo, Ilaria Pili, Chiara Rosignoli, Department of Pediatric Hematology/Oncology, Cell and Gene Therapy, IRCCS Bambino Gesù Children’s Hospital, Rome, Italy
- Francesca Bonifazi, Enrico Maffini, Ematologia, and Tiziana Lazzarotto, Microbiologia, IRCCS Azienda Ospedaliero–Universitaria di Bologna and Department of Medical and Surgical Sciences, University of Bologna, Italy
- Ilaria Cutini, SOD Terapie Cellulari e Medicina Trasfusionale and Nunziata Ciccone, SOD Microbiologia e Virologia, Azienda Ospedaliera Universitaria Careggi AOUC, Florence
- Michele Malagola, Domenico Russo, Hematology, Program of Cellular Therapies and Research in Hematology, and Arnaldo Caruso, Institute of Microbiology, Department of Molecular and Translational Medicine, University of Brescia, ASST Spedali Civili, Brescia
- Stefania Bramanti, Jacopo Mariotti, Department of Oncology/Hematology, IRCCS Humanitas Research Hospital, Rozzano, Milan
- Sofia Zompi, Alessandro Busca, Chiara Maria Della Casa, Luisa Giaccone, Irene Dogliotti, SS Trapianto Allogenico Cellule Staminali, and Antonio Curtone, SC Microbiologia e Virologia Universitaria, Dipartimento Scienze della Sanità Pubblica e Pediatrica, Università Torino, AOU Città della Salute e della Scienza di Torino, Turin
- Angelo Michele Carella, Dalila Salvatore, Daniela Valente, Francesco D’Agostino, Emanuela Merla, UOC Ematologia e Centro Trapianti CSE and Annamaria Calvo, UOSD Microbiologia e Virologia, Fondazione IRCCS Casa Sollievo della Sofferenza, San Giovanni Rotondo
- Alessandra Carotti, Loredana Ruggeri, Programma Trapianto, SC Ematologia, and Antonella Mencacci, SC Microbiologia, Azienda Ospedale Università di Perugia
- Francesco Onida, Giorgia Saporiti, Maria Goldaniga, Francesca Cavallaro Centro Trapianti di Midollo, and Patrizia Bono, Annapaola Callegaro, Laboratorio di Virologia, Fondazione IRCCS Cà Granda, Ospedale Maggiore Policlinico, Milano
- Luca Castagna, Roberto Bono, BMT Unit, AOR Villa Sofia Cervello, Palermo
- Elisabetta Terruzzi, Matteo Pama, Diego Bonardi, Fondazione IRCCS San Gerardo dei Tintori, Monza
- Adriana Vacca, Eugenia Piras, Clara Targhetta, SC Ematologia e CTMO P.O. and Annamaria Canetto, Laboratorio di Patologia Clinica, A. Businco ARNAS G Brotzu Cagliari
- Amelia Rinaldi Ematologia e CTMO and Alessia Bressan, UO Virologia, Azienda Ospedaliero–Universitaria di Parma, Parma
- Irene Maria Cavattoni, Davide Nappi, Ematologia e Centro Trapianti di Midollo, Comprensorio Sanitario di Bolzano, Azienda Sanitaria Alto Adige, Bolzano
- Alessandra Picardi, Cira Riccardi, Serena Marotta, Transplant Program and Tiziana Acione, Infectivology, AORN Cardarelli, Naples
- Maura Faraci, Gianluca Dell’Orso and Eddi Di Marco, Molecular Diagnostics, Analysis Laboratory, Hematopoietic Stem Cell Transplantation Unit, Department of Hemato-Oncology, IRCCS Istituto G. Gaslini, Genoa, Italy
- Francesca Compagno, Oncoematologia Pediatrica, Fondazione IRCCS Policlinico San Matteo, Pavia
- Edoardo Benedetti, Azienda Ospedaliera Universitaria Pisana–U.O. Ematologia Universitaria, Programma congiunto trapianto CSE e terapia cellulare, Pisa
- Antonio Maria Risitano, AORN S. Giusppe Moscati Avellino, Avellino
- Alessandro Spina, Domenico Pastore Ematologia con Trapianto, Brindisi
- Francesca Patriarca, Giampaolo Vianello, Martina Pucillo, Clinica Ematologica ed Unità Terapie Cellulari, and Corrado Pipan, Laboratorio di Sanità Pubblica, Azienda Sanitaria Universitaria Friuli Centrale, DMED, Università di Udine, Udine
- Salvatore Leotta Programma di Trapianto Emopoietico and Guido Scalia, Virologia, Azienda Ospedaliera Policlinico di Catania, Catania
- Andrea Gilioli, Angela Cuoghi, Paola Bresciani, Valeria Pioli, Andrea Masserotti, Mario Luppi SC Ematologia–Unità Trapianti di CSE, and William Gennary, SDD di Microbiologia e Virologia Molecolare, AOU di Modena, Modena
- Federico Stella, Università degli Studi di Milano, Milan
- Anna Mele, Eleonora Prete, Department of Hematology and Bone Marrow Transplant, Hospital Card. G. Panico, Tricase
- Tamara Belotti, Arcangelo Prete, Riccardo Masetti Onco-Hematology Unit, and Tiziana Lazzarotto Microbiology Unit, IRCCS Azienda Ospedaliero–Universitaria di Bologna, Bologna
- Annalisa Natale, UOS Terapia Intensiva Ematologica, Dipartimento Oncologico Ematologico and Paolo Fazii, UOC Microbiologia e Virologia Clinica a valenza regionale, Ospedale Civile, Pescara
- Cristina Skert UOC Ematologia and Mosè Favarato, laboratorio di Biologia Molecolare, Ospedale dell’Angelo Venezia Mestre
- Roberto Sorasio, Nicola Mordini, Laura Bertolotti, Antonella Maffè SC Ematologia, AO S Croce e Carle Cuneo
- Simone Cesaro, Alice Giacomazzi, Oncoematologia Pediatrica, and Davide Gibellini, Virologia Ospedale Donna Bambino, Azienda Ospedaliera Universitaria Integrata, Verona
- Piero Galieni, Department of Hematology and Stem Cell Transplantation Unit C. e G, Mazzoni Hospital, Ascoli Piceno
- Attili Olivieri, Clinica di Ematologia and Stefano Menzo, Virologia AOU delle Marche, Ancona
- Vincenzo Federico, Nicola Di Renzo, Annarita Messa, Giulia Campagna, Hematology and Transplant Unit, and Laura Lupo Microbiology, Vito Fazzi Hospital, Lecce
- Luca Facchini, Katia Codeluppi, Annalisa Imovilli, Melissa Campanelli Ematologia, and Elisa Galassi, Malattie Infettive, Azienda USL–IRCCS di Reggio Emilia, Reggio Emilia
- Carmine Selleri, Bianca Serio, Hematology and Transplant Unit and Emilia Vaccaro, Molecular Biology, AOU San Giovanni di Dio e Ruggi d’Aragona, Department of Medicine and Surgery, University of Salerno
- Franca Fagioli, Francesco Saglio, Valeria Ceolin, Oncoematologia Pediatrica and Ospedale Infantile Regina Margherita and Cristina Costa, Microbiologia Universitaria, AOU Città della Salute e della Scienza di Torino, Università degli Studi di Torino Turin
- Fabiana Cacace, UOC TCE e Terapie Cellulari–AORN Santobono Pausilipon, Naple

## References

[1] Ljungman P, de la Camara R, Robin C, et al Guidelines for the management of cytomegalovirus infection in patients with haematological malignancies and after stem cell transplantation from the 2017 European Conference on Infections in Leukaemia (ECIL 7). Lancet Infect Dis 2019; 19:e260–72. 31153807 10.1016/S1473-3099(19)30107-0

[2] Girmenia C, Lazzarotto T, Bonifazi F, et al Assessment and prevention of cytomegalovirus infection in allogeneic hematopoietic stem cell transplant and in solid organ transplant: a multidisciplinary consensus conference by the Italian GITMO, SITO, and AMCLI societies. Clin Transplant 2019; 33:e13666. 31310687 10.1111/ctr.13666

[3] Teira P, Battiwalla M, Ramanathan M, et al Early cytomegalovirus reactivation remains associated with increased transplant-related mortality in the current era: a CIBMTR analysis. Blood 2016; 127:2427–38. 26884374 10.1182/blood-2015-11-679639 PMC4874224

[4] Green ML, Leisenring W, Xie H, et al Cytomegalovirus viral load and mortality after haemopoietic stem cell transplantation in the era of pre-emptive therapy: a retrospective cohort study. Lancet Haematol 2016; 3:e119–27. 26947200 10.1016/S2352-3026(15)00289-6 PMC4914379

[5] Nichols WG, Corey L, Gooley T, Davis C, Boeckh M. High risk of death due to bacterial and fungal infection among cytomegalovirus (CMV)-seronegative recipients of stem cell transplants from seropositive donors: evidence for indirect effects of primary CMV infection. J Infect Dis 2002; 185:273–82. 11807708 10.1086/338624

[6] Boeckh M, Nichols WG. The impact of cytomegalovirus serostatus of donor and recipient before hematopoietic stem cell transplantation in the era of antiviral prophylaxis and preemptive therapy. Blood 2004; 103:2003–8. 14644993 10.1182/blood-2003-10-3616

[7] Schmidt-Hieber M, Labopin M, Beelen D, et al CMV serostatus still has an important prognostic impact in de novo acute leukemia patients after allogeneic stem cell transplantation: a report from the Acute Leukemia Working Party of EBMT. Blood 2013; 122:3359–64. 24037724 10.1182/blood-2013-05-499830

[8] Ljungman P, Brand R, Hoek J, et al Infectious diseases working party of the European group for blood and marrow transplantation. Donor cytomegalovirus status influences the outcome of allogeneic stem cell transplant: a study by the European Group for Blood and Marrow Transplantation. Clin Infect Dis 2014; 59:473–81. 24850801 10.1093/cid/ciu364

[9] Marty FM, Ljungman P, Chemaly RF, et al Letermovir prophylaxis for cytomegalovirus in hematopoietic-cell transplantation. N Engl J Med 2017; 377:2433–44. 29211658 10.1056/NEJMoa1706640

[10] Vyas A, Raval AD, Kamat S, LaPlante K, Tang Y, Chemaly RF. Real-world outcomes associated with letermovir use for cytomegalovirus primary prophylaxis in allogeneic hematopoietic cell transplant recipients: a systematic review and meta-analysis of observational studies. Open Forum Infect Dis 2022; 10:ofac687. 36726548 10.1093/ofid/ofac687 PMC9879759

[11] von Elm E, Altman DG, Egger M, et al The Strengthening the Reporting of Observational Studies in Epidemiology (STROBE) statement: guidelines for reporting observational studies. J Clin Epidemiol 2008; 61:344–9. 18313558 10.1016/j.jclinepi.2007.11.008

[12] Ljungman P, Boeckh M, Hirsch HH, et al Definitions of cytomegalovirus infection and disease in transplant patients for use in clinical trials. Clin Infect Dis 2017; 64:87–91. 27682069 10.1093/cid/ciw668

[13] Boeckh M, Ljungman P. How we treat cytomegalovirus in hematopoietic cell transplant recipients. Blood 2009; 113:5711–9. 19299333 10.1182/blood-2008-10-143560 PMC2700312

[14] Ljungman P . Molecular monitoring of viral infections after hematopoietic stem cell transplantation. Int J Hematol 2010; 91:596–601. 20414752 10.1007/s12185-010-0570-4

[15] Cesaro S, Ljungman P, Tridello G, et al New trends in the management of cytomegalovirus infection after allogeneic hematopoietic cell transplantation: a survey of the Infectious Diseases Working Party of EBMT. Bone Marrow Transplant 2023; 58:203–8. 36396949 10.1038/s41409-022-01863-8 PMC9672643

[16] Zamora D, Duke ER, Xie H, et al Cytomegalovirus-specific T-cell reconstitution following letermovir prophylaxis after hematopoietic cell transplantation. Blood 2021; 138:34–43. 33657225 10.1182/blood.2020009396 PMC8493975

[17] Gabanti E, Borsani O, Colombo AA, et al Human cytomegalovirus-specific T-cell reconstitution and late-onset cytomegalovirus infection in hematopoietic stem cell transplantation recipients following letermovir prophylaxis. Transplant Cell Ther 2022; 28:211.e1–9. 10.1016/j.jtct.2022.01.008 35042012

[18] Lauruschkat CD, Muchsin I, Rein A, et al CD4+ T cells are the major predictor of HCMV control in allogeneic stem cell transplant recipients on letermovir prophylaxis. Front Immunol 2023; 14:1148841. 37234158 10.3389/fimmu.2023.1148841 PMC10206124

[19] Zavaglio F, Vitello D, Bergami F, et al Human cytomegalovirus (HCMV)-specific T-cell response after letermovir prophylaxis is predictive for subsequent HCMV reactivation in haematopoietic stem cell transplant recipients. J Clin Virol 2023; 165:105519. 37321150 10.1016/j.jcv.2023.105519

[20] Solano C, Giménez E, Albert E, Piñana JL, Navarro D. Immunovirology of cytomegalovirus infection in allogeneic stem cell transplant recipients undergoing prophylaxis with letermovir: a narrative review. J Med Virol 2023; 95:e29005. 37526411 10.1002/jmv.29005

[21] Russo D, Schmitt M, Pilorge S, et al Efficacy and safety of extended duration letermovir prophylaxis in recipients of haematopoietic stem-cell transplantation at risk of cytomegalovirus infection: a multicentre, randomised, double-blind, placebo-controlled, phase 3 trial. Lancet Haematol 2024; 11:e127–35. 38142695 10.1016/S2352-3026(23)00344-7

[22] Febres-Aldana A, Khawaja F, Morado-Aramburo O, et al Mortality in recipients of allogeneic haematopoietic cell transplantation in the era of cytomegalovirus primary prophylaxis: a single-centre retrospective experience. Clin Microbiol Infect 2024; 30:803–9. 38460821 10.1016/j.cmi.2024.03.001 PMC12265989

